# Identification of metabolism‐associated molecular subtype in ovarian cancer

**DOI:** 10.1111/jcmm.16907

**Published:** 2021-09-15

**Authors:** Xiaona Liu, Aoshen Wu, Xing Wang, Yunhe Liu, Yiang Xu, Gang Liu, Lei Liu

**Affiliations:** ^1^ Institutes of Biomedical Sciences Fudan University Shanghai China; ^2^ School of Basic Medical Sciences Fudan University Shanghai China; ^3^ State Key Laboratory of Neuroscience Institute of Neuroscience Center for Excellence in Brain Science and Intelligence Technology Chinese Academy of Sciences Shanghai China; ^4^ University of Chinese Academy of Sciences Beijing China

**Keywords:** immune, metabolic genes, ovarian cancer, prognosis, subtype

## Abstract

Ovarian cancer (OC) is the most lethal gynaecological cancer with genomic complexity and extensive heterogeneity. This study aimed to characterize the molecular features of OC based on the gene expression profile of 2752 previously characterized metabolism‐relevant genes and provide new strategies to improve the clinical status of patients with OC. Finally, three molecular subtypes (C1, C2 and C3) were identified. The C2 subtype displayed the worst prognosis, upregulated immune‐cell infiltration status and expression level of immune checkpoint genes, lower burden of copy number gains and losses and suboptimal response to targeted drug bevacizumab. The C1 subtype showed downregulated immune‐cell infiltration status and expression level of immune checkpoint genes, the lowest incidence of BRCA mutation and optimal response to targeted drug bevacizumab. The C3 subtype had an intermediate immune status, the highest incidence of BRCA mutation and a secondary optimal response to bevacizumab. Gene signatures of C1 and C2 subtypes with an opposite expression level were mainly enriched in proteolysis and immune‐related biological process. The C3 subtype was mainly enriched in the T cell‐related biological process. The prognostic and immune status of subtypes were validated in the Gene Expression Omnibus (GEO) dataset, which was predicted with a 45‐gene classifier. These findings might improve the understanding of the diversity and therapeutic strategies for OC.

## 1 INTRODUCTION

Ovarian cancer (OC) is the most lethal gynaecological cancer with 313,959 new cases, and 207,252 deaths reported worldwide in 2020; also, it represents the eighth most commonly diagnosed cancer among women in the world.^1^, ^2^ About 75% of patients are diagnosed at an advanced stage because of the asymptomatic nature of early‐staged OC.^1^ Advanced‐stage patients have a 5‐year relative survival rate of 29%, while this rate was 92% for early‐stage disease.^3^ Despite improvements in surgery, chemotherapy, radiotherapy, targeted therapy and endocrine therapy, the current treatment effect of OC is still not satisfactory. Therefore, further understanding of the biology of OC and effective treatment measures is required to improve the clinical outcome of patients with OC.

The anomalous metabolism in cancer was gradually found and regarded as a potential therapeutic approach. Metabolic reprogramming was an emerging hallmark of cancer in 2011^4^ and has been extensively investigated over the past decade. Cancer cells can reprogram their glucose metabolism even in the presence of oxygen, leading to a state termed as ‘aerobic glycolysis’.^4^ Besides, the lipid‐related pathway is a common and important mechanism in cancer cells for supplying energetic expenditure and membrane synthesis.^5^ This is also applicable to OC cells, and most studies have reported the abnormal lipid metabolism in OC.^6^, ^7^, ^8^ Although lipids and some subclasses of nutritional lipids may be associated with epithelial ovarian carcinoma (EOC) risk, the metabolism of lysophosphatidic acid (LPA) and arachidonic acid (AA) emerges as an important signalling network in EOC.^8^ Emerging evidence suggests that tumour cells have much more complex metabolic requirements than previously reported, and metabolic reprogramming is an important strategy of cancer therapy.^9^ Therefore, the molecular subtype of OC based on metabolomic signatures may have great clinical significance.

In this study, we used the expression data of metabolism‐relevant genes from the Cancer Genome Atlas (TCGA) database and identified three molecular subtypes of OC, namely C1, C2 and C3. International Cancer Genome Consortium (ICGC) and GEO datasets were used for validation. We also evaluated the prognostic value, transcriptome features, metabolic signatures, immune‐cell infiltration and drug sensitivity of the OC subtypes. Finally, a 45‐gene classifier was developed and validated.

## 2 MATERIALS AND METHODS

### 2.1 Data source and processing

Three data repositories, including TCGA (https://portal.gdc.cancer.gov/), ICGC (https://daco.icgc.org/) and GEO (https://www.ncbi.nlm.nih.gov/geo/), were used to obtain required data for OC. RNA sequencing data (raw counts and Fragments per Kilobase Million [FPKM]) of 373 OC human samples with prognostic data were retrieved from the TCGA‐OV cohort using R package ‘TCGAbiolink’. RNAseq data (FPKM) of 81 OC samples with clinical information were downloaded from the ICGC database. The FPKM data were transformed into transcripts per kilobase million (TPM) values and then log2 transformed for subsequent analysis. Additional raw microarray data of 260 OC samples from GSE32062 (GPL6480) were downloaded and processed by a robust multi‐array average (RMA) algorithm using R package ‘GEOquery’. Gene somatic mutation data (MAF files) and copy number data of the TCGA cohort were downloaded from UCSC Xena (https://xena.ucsc.edu/). The GSE140082 dataset including the bevacizumab response results was included in the analysis to investigate targeted drug response.

### 2.2 Identification of OC subtypes

Previously published 2752 metabolism‐relevant genes encoding known human metabolic enzymes and transporters^10^ were used as candidate genes for identifying subtypes based on non‐negative matrix factorization (NMF) clustering.^11^ A filtering step was performed on the TCGA cohort before NMF clustering. Genes with a high median absolute deviation (MAD) value (MAD >0.5) across all samples and significant prognostic value (*p* < 0.05, Cox regression using R packages ‘survival’) were used for NMF clustering. Subsequently, an unsupervised NMF clustering method was used to cluster samples of TCGA and ICGC cohorts using the R package ‘NMF’.^12^ The value of *k* where the magnitude of the cophenetic correlation coefficient began to fall was chosen as the optimal number of clusters.^13^ A class mapping (SubMap) analysis (Gene Pattern, https://www.genepattern.org/) was used to evaluate the similarity of the subtypes identified in the two aforementioned datasets based on their expression profiles.^14^ T‐distributed stochastic neighbour embedding (t‐SNE)^15^ (R packages ‘Rtsne’) was also used to validate the subtype assignments with the mRNA expression data of the aforementioned metabolic genes.

### 2.3 Differential analysis and function enrichment analysis

The differentially expressed genes (DEGs) among OC subtypes were identified using the *limma* package in R on the TCGA‐OV raw count data. Absolute log2FC >1.5 and adjusted *p* < 0.01 were thresholds for DEGs. Subsequently, Gene Set Enrichment Analysis (GSEA) software^16^ was used for KEGG pathway enrichment analysis with the expression profile of TCGA‐OV. DAVAD^17^, ^18^ website (https://david.ncifcrf.gov/home.jsp) was used for GO BP (Gene Ontology, Biological Process) enrichment analysis using the expression profiles of DEGs.

### 2.4 Metabolism‐relevant pathway gene signature score using gene set variation analysis

Gene set variation analysis (GSVA), a nonparametric and unsupervised gene set enrichment method, can estimate the score of a certain pathway or signature based on transcriptomic data.^19^ The 114 metabolism‐relevant pathway gene signatures were acquired from previously published studies.^20^ Each sample received 114 scores corresponding to 114 metabolism pathway signatures using the R package ‘GSVA’. Subsequently, differential analysis was conducted based on 114 metabolism scores using the ‘limma’ package in the R software, and adjusted *p* < 0.05 was used for identifying differentially signatures.

### 2.5 Estimation of immune infiltration

Immune scores and stromal scores were calculated using the ESTIMATE algorithm (R package ‘estimate’), which could infer the fraction of stromal and immune cells in tumour samples.^21^ We evaluated the abundance of two immune cell populations (T cells and myeloid dendritic cells) and two nonimmune stromal cell population (cancer‐associated fibroblasts and endothelial cells) using a microenvironment cell population‐counter (MCP‐counter)^22^ in the Timer website. Besides, a single‐sample GSEA (ssGSEA) algorithm in the *GSVA* R package was used to estimate the immune cell infiltration of 28 immune cell populations.^23^

### 2.6 Generation of classifier and performance validation

The top 15 DEGs with the largest log2FC value (only DEGs with log2FC >0 were chosen) in each subtype were selected to develop the prediction model, and a 45‐gene classifier was generated. The subtype prediction was conducted based on the 45‐gene classifier on the GSE32062 dataset using nearest template prediction (NTP) algorithm (*CMScaller* R package). Subsequently, the prognosis and immune‐cell infiltration features of predicted subtypes were analysed to evaluate the performance of classifier and stability of subtypes.

### 2.7 Response prediction of each subtype for targeted therapy

The response of bevacizumab to each subtype of the TCGA cohort was predicted based on data derived from the GSE140082 dataset using the SubMap algorithm.

### 2.8 Other statistical analyses

R software (4.0.2, https://www.r‐project.org/) was used for statistical analysis. Log‐rank test and Kaplan‐Meier methods were used to analyse the prognostic differences in different subtypes. The unpaired Student's *t* test was used to compare the two groups with normally distributed variables, while the Mann‐Whitney *U* test was used to compare two groups with the non‐normally distributed variables. All scripts were placed on GitHub (https://github.com/Nanahaha/Article_script).

## 3 RESULTS

### 3.1 NMF identified three molecular subtypes in OC

**FIGURE 1:**
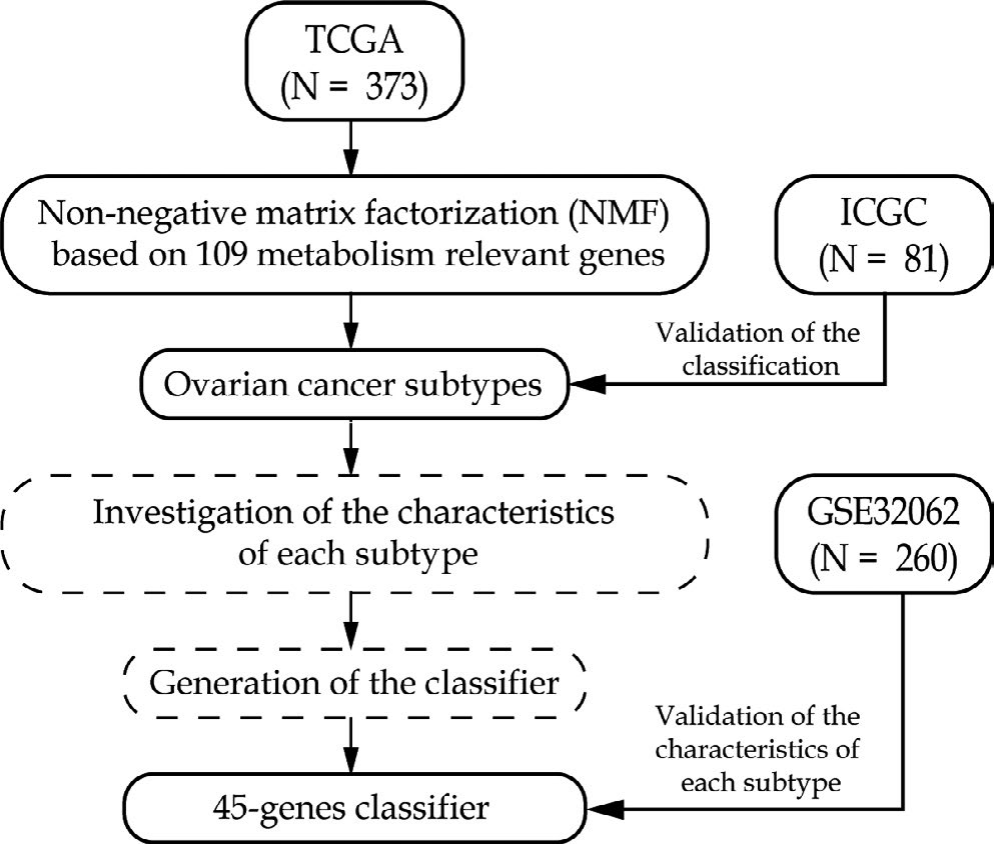
The workflow of the study

**FIGURE 2:**
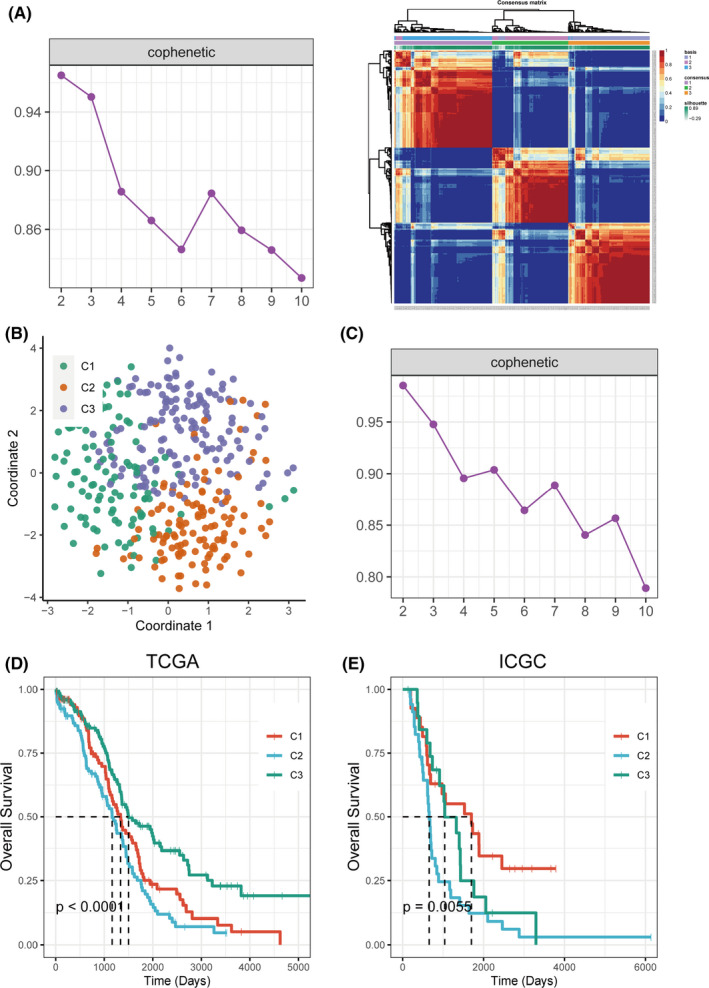
Identification of subtypes using NMF consensus clustering based on the metabolism‐associated genes. (A) NMF clustering using 109 metabolism‐associated genes in TCGA cohort. Cophenetic correlation coefficient for *k* = 2–10 was shown. The consensus matrix heatmap for *k* = 3 was shown. (B) t‐SNE analysis scatter plot based on 109 metabolism‐associated genes. (C) Cophenetic correlation coefficient of NMF clustering for *k* = 2–10 in ICGC cohort was shown. (D) KM curves showed prognostic relationship of three subtypes in TCGA and ICGC cohort, respectively; the *p*‐value was calculated using the log‐rank test, by comparing the overall survival of three subtypes

The study workflow is shown in Figure 1. Previously reported 2752 metabolism‐relevant genes were chosen as the basis of NMF analysis. After screening, the expression profile of 109 metabolism‐relevant genes was used to identify molecular subtypes using NMF consensus clustering. As shown in Figure 2A, the optimal number of clusters was *k* = 3 according to the cophenetic correlation coefficients. When *k* = 3, the consensus matrix heatmap still had clear boundaries, suggesting stable and robust clustering for the samples (Figure S1A). T‐SNE was performed on the expression profile of the 109 metabolism‐relevant genes to validate the assignment, and the three subtypes were clustered separately in a two‐dimensional t‐SNE distribution patterns (Figure 2B). In addition, the ICGC dataset with 81 OC samples was used to validate the classification based on 109 metabolism‐relevant genes and NMF consensus clustering algorithm. Consistently, the results of the ICGC dataset also revealed that of the number of optimized subtypes was also 3, suggesting the robustness and stability of the three subtypes (Figure 2C and Figure S1B). A SubMap analysis was conducted to analyse the correlation of subtypes between TCGA and ICGC datasets. The result showed that C1, C2 and C3 subtypes in the TCGA dataset highly correlated with corresponding subtypes in the ICGC dataset, suggesting the robustness of subtyping across cohorts (Figure S2).

The differences in survival among subtypes were analysed by the Kaplan‐Meier method (Figure 2D,E). Significant differences in overall survival among subtypes were found in both the TCGA dataset (*p* < 0.0001) and the ICGC dataset (*p* = 0.0055). The overall survival of the C2 subtype was the worst in both datasets.

### 3.2 Transcriptomic characteristics and metabolism‐related pathway characteristics of OC subtypes

Subtype‐specific genes (DEGs) were identified with the thresholds of the false discovery rate (FDR) <0.01 and |log2FC| >1.5. A total of 520, 292 and 43 DEGs were identified in C1, C2 and C3 (Table S1), respectively. Subsequently, the GSEA pathway and GO BP enrichment analyses were performed (Figure S3, Figure S4 and Table S2). Most DEGs of the C1 subtype were downregulated, while those of the C2 subtype were upregulated. Both C1 and C2 subtypes were significantly enriched in the ‘cytokine cytokine receptor interaction’ and ‘hematopoietic cell lineage’ pathway, but had the opposite enrichment status. Consistent with this, the GO BP enrichment analysis showed a similar pattern. Gene signatures of C1 and C2 subtypes were mainly enriched in proteolysis, immune‐related biological process and Fc receptor‐related process, but the regulatory status was also opposite depending on the relative expression status of the genes in GO terms. The C3 subtype was mainly enriched in the phosphorylation pathway and T cell‐related biological process but with no statistical significance.

Since the subtyping was based on metabolism‐relevant genes, the correlation between OC subtypes and metabolism pathways was investigated. The metabolic pathways were retrieved from a previous published study.^20^ The GSVA algorithm was used to score each sample targeted to 114 metabolism pathways. Then, the difference in metabolic pathways between one subtype and the other subtypes was analysed using the *limma* package. The FDR <0.05 was used as the threshold for identifying significantly different metabolic pathways of each subtype. The heatmap of metabolism pathway signatures of the C2 subtype is shown in Figure S5A. The C2 subtype had significantly increased glycan biosynthesis‐related pathways and several carbohydrate metabolism‐ and lipid metabolism‐related pathways (eg glycerophospholipid, glycosphingolipid and sphingolipid metabolism) (Figure S5B). Aldosterone, ketone, steroid hormone and testosterone metabolism had the lowest scores in the C2 subtype (Figure S5B).

### 3.3 Correlation of OC subtypes with the immune status

**FIGURE 3:**
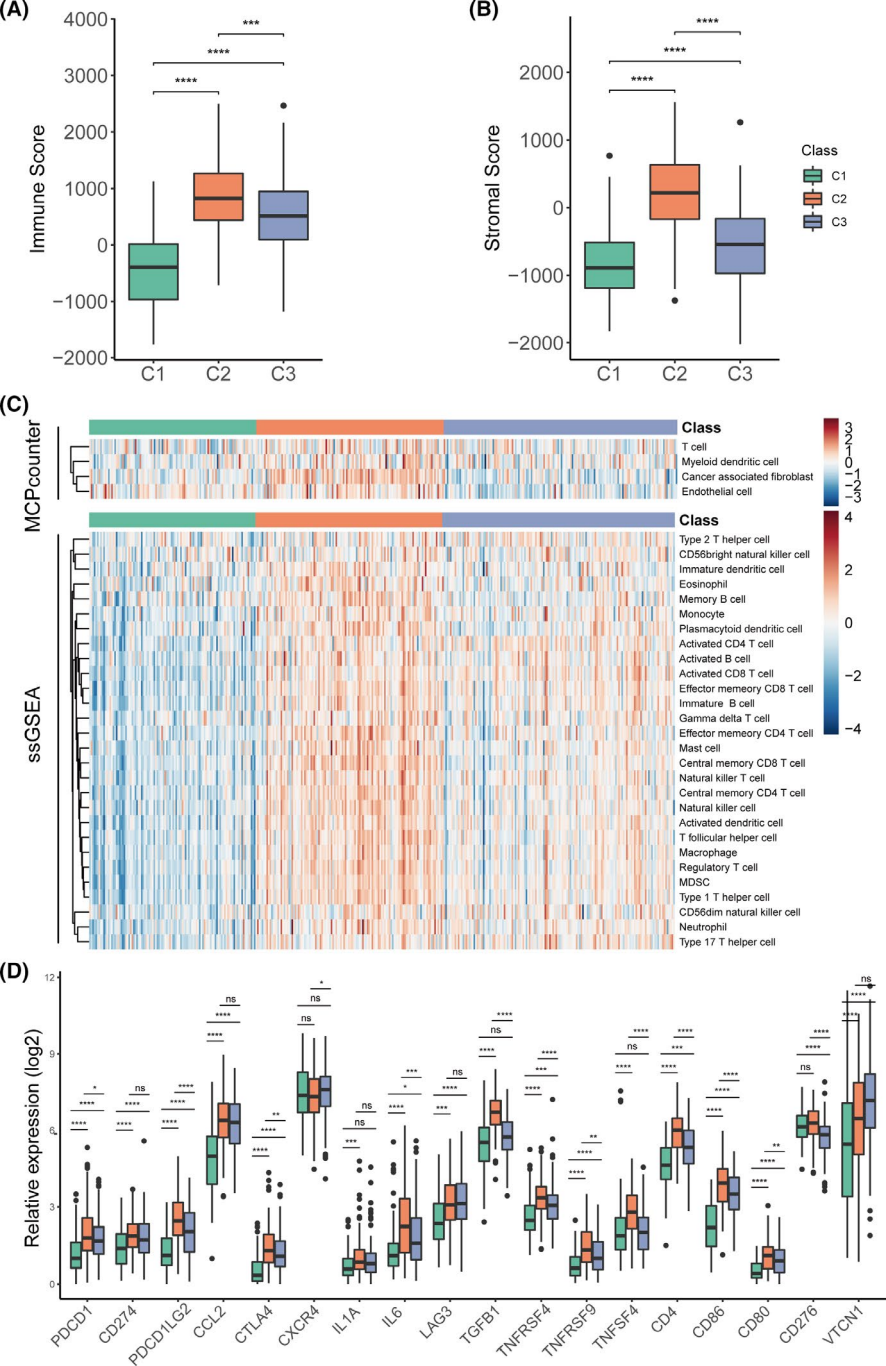
Immune characteristics of the three subtypes in the TCGA‐OV cohort. Boxplot of immune score (A) and stromal score (B) from ESTIMATE of three subtypes. (B) Heatmap describing the abundance of immune and stromal cell population in C1, C2 and C3. (D) Expression level of 18 immune checkpoint genes in three OC subtypes. The statistical difference was compared by wilcox.test, and adjusted by ‘holm’ method (ns represents no significance, *p < 0.05, **p < 0.01, ***p < 0.001, ****p < 0.0001)

Immune therapy is a hotspot of cancer therapy at present. Thus, the immune characteristics of the three subtypes were explored. Firstly, the tumour purity of each subtype was evaluated using the ESTIMATE algorithm (Figure 3A,B), which is used to estimate stromal and immune cells in malignant tumour tissues with expression data. The results showed that the C2 subtype had significantly increased immune and stromal scores than C1 and C3 subtypes, while the C1 subtype had the lowest scores. These results suggested that the subtypes of OC might have different immune responses.

Subsequently, the immune‐cell infiltration score was analysed using the MCP‐counter algorithm and the ssGSEA algorithm. The infiltration scores of 32 immune cell types were evaluated (Figure 3C, Figure S6). Consistent with previous results, the C2 subtype had a significantly increased immune cell infiltration level (except activated CD4 T cells, activated CD8 T cells, CD56bright natural killer cells, CD56dim natural killer cells and type 2 T helper cells), while the C1 subtype had a significantly decreased immune cell infiltration (except memory B cells). Further, the C3 subtype had a moderate immune cell infiltration (except activated CD4 T cells, activated CD8 T cells, CD56bright natural killer cells, CD56dim natural killer cells, type 2 T helper cells and memory B cells; Figure 3C, Figure S6).

The immune checkpoint is the critical target for immune therapy. The expression of 18 immune checkpoints (PDCD1, CD274, PDCD1LG2, CCL2, CTLA4, CXCR4, IL1A, IL6, LAG3, TGFB1, TNFRSF4, TNFRSF9, TNFSF4, CD4, CD86, CD80, CD276 and VTCN1) was analysed among the three subtypes (Figure 3D). The results showed that the C2 subtype had significantly increased expression of most checkpoint genes except CXCR4, LAG3 and VTCN1, while the C1 subtype had significantly decreased expression except CXCR4 and CD276 (Figure 3D).

Collectively, the three subtypes of OC showed significantly differences in the immune status.

### 3.4 Mutations and copy number aberrations of OC subtypes

**FIGURE 4:**
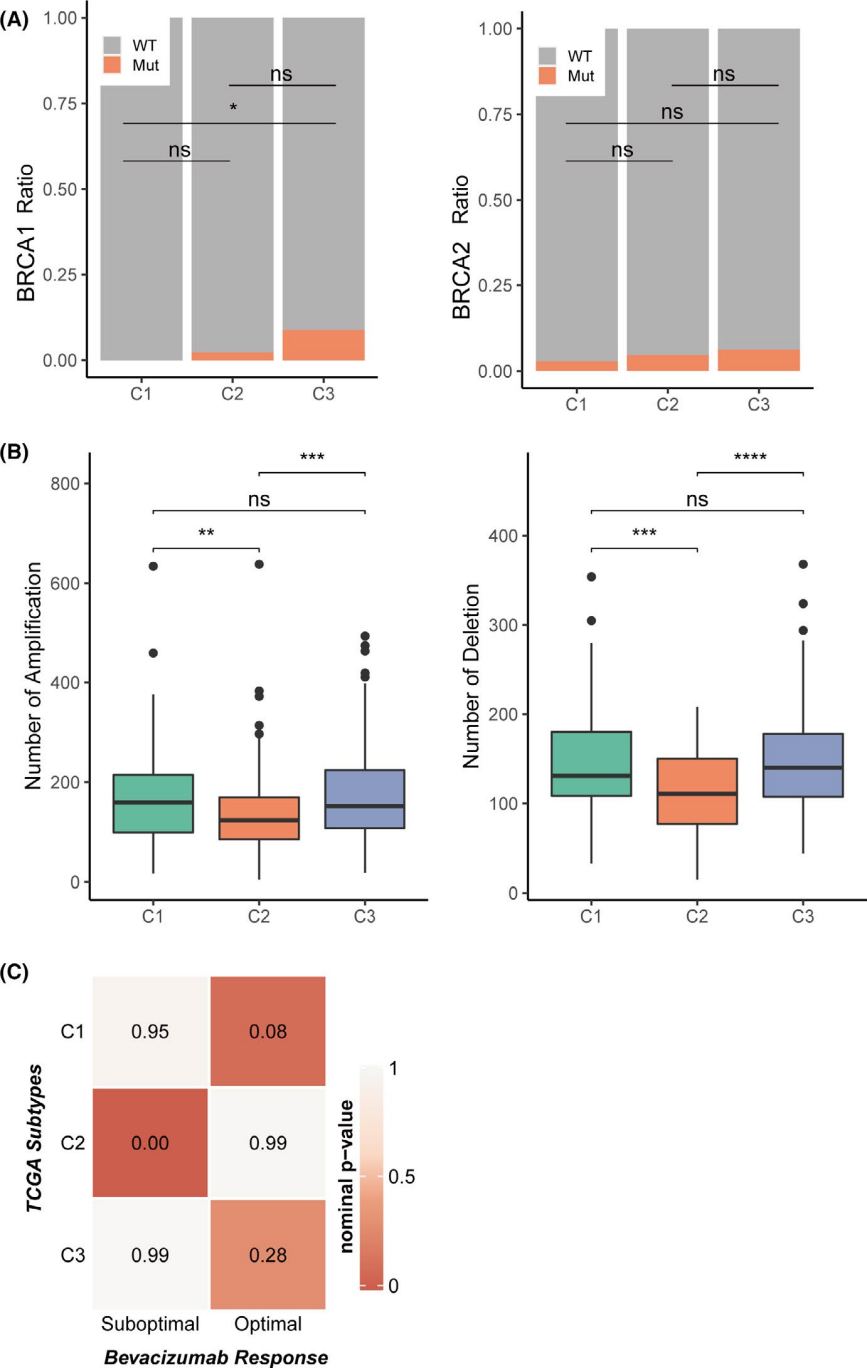
The association between three subtypes and mutations, copy number aberrations and targeted drug response in TCGA‐OV cohort. (A) BRCA1 and BRCA2 mutation type/wild‐type ratio in three subtypes. (C) Boxplot of number of copy number aberrations. (D) Correlation between three subtypes and bevacizumab response. Between three subtypes. The statistical difference was compared by wilcox.test, and adjusted by ‘holm’ method (ns represents no significance, *p < 0.05, **p < 0.01, ***p < 0.001, ****p < 0.0001)

BRCA is a tumour suppressor gene that plays an important role in regulating cell replication, repair of DNA damage and normal cell growth.^24^ BRCA mutation leads to the loss of function of tumour growth inhibition.^25^ We analysed the relationship between BRCA1 and BRCA2 mutations and the three subtypes (Figure 4A). The results showed that the mutation rate of C3 BRCA1 was significantly higher than that of C1 (Fisher's test, *p* = 0.025). The mutation rate of C3 BRCA2 was higher than C1/C2, but with no statistical significance. In terms of somatic copy number aberrations, C2 showed a lower burden of both gains and losses compared with other subtypes, while no statistically significant difference in gain and loss was found between C1 and C3 (Figure 4B).

### 3.5 Correlation of OC subtypes with the sensitivity of targeted drugs

Targeted drugs are an effective approach to cancer therapy. Bevacizumab, a monoclonal antibody, targets the vascular endothelial growth factor (VEGF) pathway and inhibits tumour blood vessel growth. Thus, this study evaluated the correlation between bevacizumab responses and OC subtypes. An array expression profile including the bevacizumab response results in OC was downloaded from GEO (GSE140082). A SubMap analysis was conducted to analyse the correlation between the three subtypes and the bevacizumab response. The results showed that the C1 subtype in the TCGA dataset highly correlated with an optimal response to bevacizumab in the GSE140082 dataset, the C2 subtype was highly correlated with a suboptimal response to bevacizumab, while the C3 subtype was secondarily correlated with the optimal response to bevacizumab (Figure 4C). These results suggested that bevacizumab therapeutic strategies might have different responses to subtypes.

### 3.6 Forty‐five‐gene classifier and performance validation

**FIGURE 5:**
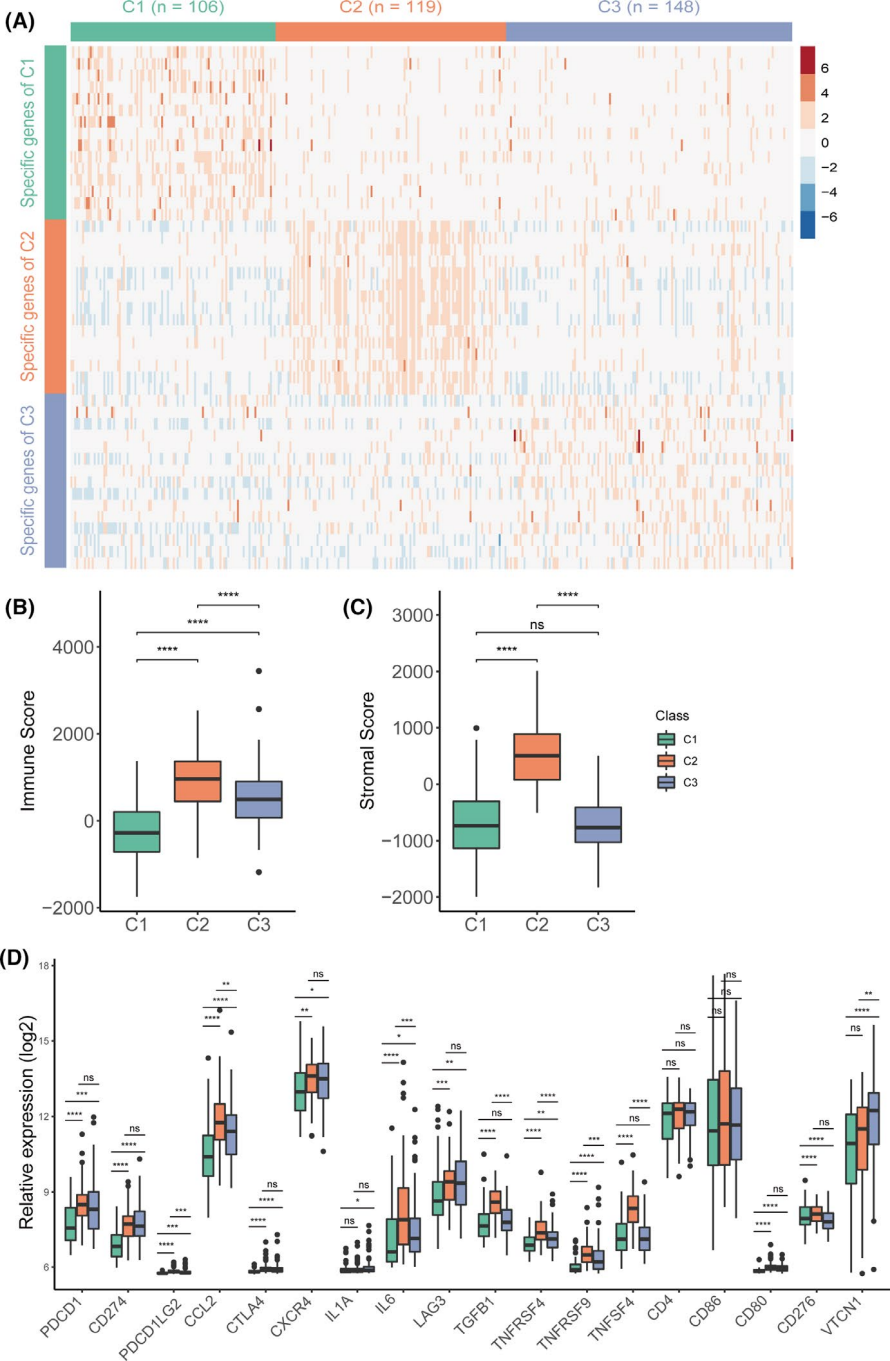
Validation of external dataset. (A) Heatmap of the expression level of the 45‐classifier in TCGA‐OV cohort. Boxplot of immune score (B) and stromal score (C) from ESTIMATE of three predicted subtypes in the validation set. (D) Expression distribution of 18 checkpoint genes in three subtypes in the validation set. The statistical difference was compared by wilcox.test, and adjusted by ‘holm’ method (ns represents no significance, *p < 0.05, **p < 0.01, ***p < 0.001, ****p < 0.0001)

The GSE32062 dataset including 260 samples was downloaded from the GEO database to validate the three subtypes further. Considering clinical application potential, the top informative subtype‐associated signature genes including top 15 genes with the largest log2FC value (>0) in each subtype were selected. The NTP algorithm was used to predict the subtype of the GSE32062 dataset using a 45‐gene classifier (Figure 5A, Table S3). Further, 63 samples were classified into the C1 subtype, 86 samples into the C2 subtype and 112 samples into the C3 subtype. We first analysed the difference in overall survival among three predicted subtypes in the GEO dataset and found that the C2 subtype had the worst survival (Figure S7). Next, we analysed the tumour purity of the predicted three subtypes. The results showed that the immune score and stromal scores were significantly higher in the C2 subtype than in other subtypes, and the immune scores were significantly lower in the C1 subtype than in other subtypes (Figure 5B). Besides, the expression distribution of 18 immune checkpoint genes was also analysed among the three subtypes. High expression of most immune checkpoint genes in the C2 subtype (Figure 5D) was observed. The results in the GSE32062 dataset were consistent with those in the TCGA dataset.

## 4 DISCUSSION

The studies on the molecular subtyping of OC were mainly based on the overall expression profile. Despite comprehensive transcriptomic information, redundancy and noise were also employed. The importance of metabolism in cancer was gradually realized.^4^ To investigate the association between OC subtypes and metabolic processes, OC subtypes were established in this study based on 2752 metabolic genes previously studied,^10^ which provided more details about the metabolomic landscape of OC.

In this study, three subtypes (C1, C2 and C3) of OC were identified based on metabolism‐relevant genes in the TCGA‐OV cohort. The robustness of the subtype number was verified in the external dataset ICGC‐OV. A classifier consisting of 45 genes was evaluated in the external dataset GSE32062. The transcriptome features, metabolic pathway signatures, prognosis, immune infiltration and targeted drug response of the three subtypes were explored. Overall, C1 and C2 subtypes showed significant differences in most molecular characteristics.

The mutation rate of BRCA1 was significantly higher in the C3 subtype compared with the other subtypes. BRCA1/2 absence or mutation increased the response sensitivity to DNA cross‐linkers (eg cisplatin), and it has been shown that cisplatin and paclitaxel combination chemotherapy benefited patients with BRCA1 mutation more,^26^ which was consistent with the fact that C3 subtype with the highest mutation rate of BRCA1 had a good prognosis in the present study. In terms of copy number aberrations, C2 patients showed a lower burden of gains and losses, suggesting the stability of the C2 genome.

Previous studies demonstrated that metabolic alterations were existed in OC;^27^ however, the metabolic heterogeneity was rarely reported. The results of the present study indicated that each subtype had unique metabolism signatures. The C2 subtype was chiefly involved in glycan biosynthesis and metabolism‐relevant signatures, carbohydrate metabolism and lipid metabolism processes. Among the signatures, glycans, which decorated all eukaryotic cell surfaces, underwent changes in structure with the onset of diseases such as cancer and inflammation. Several glycan‐based vaccines are presently undergoing clinical evaluation with some encouraging preliminary results.^28^ The expression of Sialyl‐Tn (STn), a type of O‐glycans, was increased in cancer, and the prognosis was poor in patients with cancer.^29^ Our results revealed that the C2 subtype had a high enrichment score in glycan biosynthesis and metabolism‐relevant signatures compared with the other two subtypes, suggesting that the C2 subtype might be more sensitive to glycan‐based vaccines. Lipid metabolism alteration is an important characteristic of OC.^30^ Our results showed that several lipid metabolism‐relevant signatures (eg cyclooxygenase arachidonic acid, glycerolipid, fatty acid biosynthesis, glycerophospholipid, glycosphingolipid, sphingolipid) had higher enrichment scores in the C2 subtype compared with the other subtypes. Aberrant lipid metabolic genes were found to be associated with worse clinical outcomes. For example, fatty acid synthase (FASN) is highly expressed in OC tissues and is associated with poor prognosis.^31^ The increased levels of ASAH1 and sphingosine‐1‐phosphate (S1P), which participated in sphingolipid metabolism, were associated with the poor survival of OC.^27^, ^30^ These reports might explain why the C2 subtype had the worst overall survival among the subtypes.

Previous studies suggested that immune checkpoints played a crucial role in the immune escape of cancer.^32^ The monoclonal antibodies developed for PD‐1 and its ligands as well as CTLA4 have been approved for the clinical therapy of several cancers (eg melanoma and non‐small‐cell lung carcinoma).^33^, ^34^ Thus, the difference in the expression of immune checkpoint genes among the subtypes was evaluated. The C2 subtype had the highest level immune cell infiltration. An OC subtype classification based on immune genes reported the worst prognosis in one subtype with abundant immune infiltration,^35^ which was consistent with the result of the present study. PD‐1, which was upregulated in the C2 subtype in this study, inhibits excessive immune responses and protects normal cells from immune attack.^36^ High PD‐1+ expression of lymphocytes was associated with the poor survival of OC.^37^ Regulatory CD4+ T (Treg) cells and myeloid‐derived suppressor cells (MDSCs) were also significantly enriched in C2. Consistently, regulatory CD4+ T (Treg) cells mediated homeostatic peripheral tolerance by suppressing autoreactive T cells.^38^ A previous study showed that tumour Treg cells were associated with a high death hazard and reduced survival in OC.^38^ Baert et al.^39^ reported that MDSCs, which belonged to the innate immune system, were the key drivers of immunosuppression in OC. These findings might also explain why the C2 subtype had a high degree of immune cell infiltration but a poor prognosis. Although no immune therapies are currently approved for OC,^40^ our subtyping results might provide some information for OV immune therapy.

This study first established the molecular subtypes of OC using a metabolism‐relevant gene expression profile and investigated the characteristics of subtypes. However, the study had some limitations. First, the number of metabolism‐relevant genes used to identify subtypes was small. Second, the number of genes in classifiers was also small. Third, no difference in prognosis was found in the GEO32062 validation set, but was consistent with the survival trends in the TCGA dataset. Fourth, the survival of subtypes in the TCGA and ICGC datasets was inconsistent. This might be due to the small sample size of the ICGC dataset or the inherently small difference in survival rates between subtypes. The worst survival rate for the C2 subtype was the most important result in all datasets.

## 5 CONCLUSION

This study identified three subtypes in OC using gene expression profiles of metabolism‐relevant genes through large databases of TCGA, ICGC and GEO. The three subtypes exhibited unique characteristics in terms of prognosis, transcriptome, immune and mutation statuses, and copy number. These findings may shed new light on individual therapeutic strategies for OC.

## CONFLICT OF INTEREST

The authors confirm that there are no conflicts of interest.

## AUTHOR CONTRIBUTIONS

**Lei Liu:** Funding acquisition (lead) and Supervision (lead). **Xiaona Liu:** Conceptualization (equal); Formal analysis (equal); Methodology (equal); and Writing—original draft (equal). **Aoshen Wu:** Formal analysis (equal); Software (equal); and Writing—original draft (equal). **Xing Wang:** Conceptualization (equal); Formal analysis (equal); and Writing—original draft (equal). **Yunhe Liu:** Methodology (equal). **Yiang Xu:** Investigation (equal). **Gang Liu:** Project administration (lead) and Writing—review & editing (lead).

## Supporting information

Fig S1‐7

Table S1

Table S2

Table S3

## Data Availability

Data sharing is not applicable to this article as no new data were created or analysed in this study.
