# Supplementary material for: Identification of metabolism‐associated molecular subtype in ovarian cancer
Source: J Cell Mol Med. 2021 Sep 15;25(20):9617–26. doi: 10.1111/jcmm.16907 (PMC8505839; doi:10.1111/jcmm.16907)
Supplement: Supplementary file 1 — Fig S1‐7 [file JCMM-25-9617-s003.docx]

Supplementary Materials


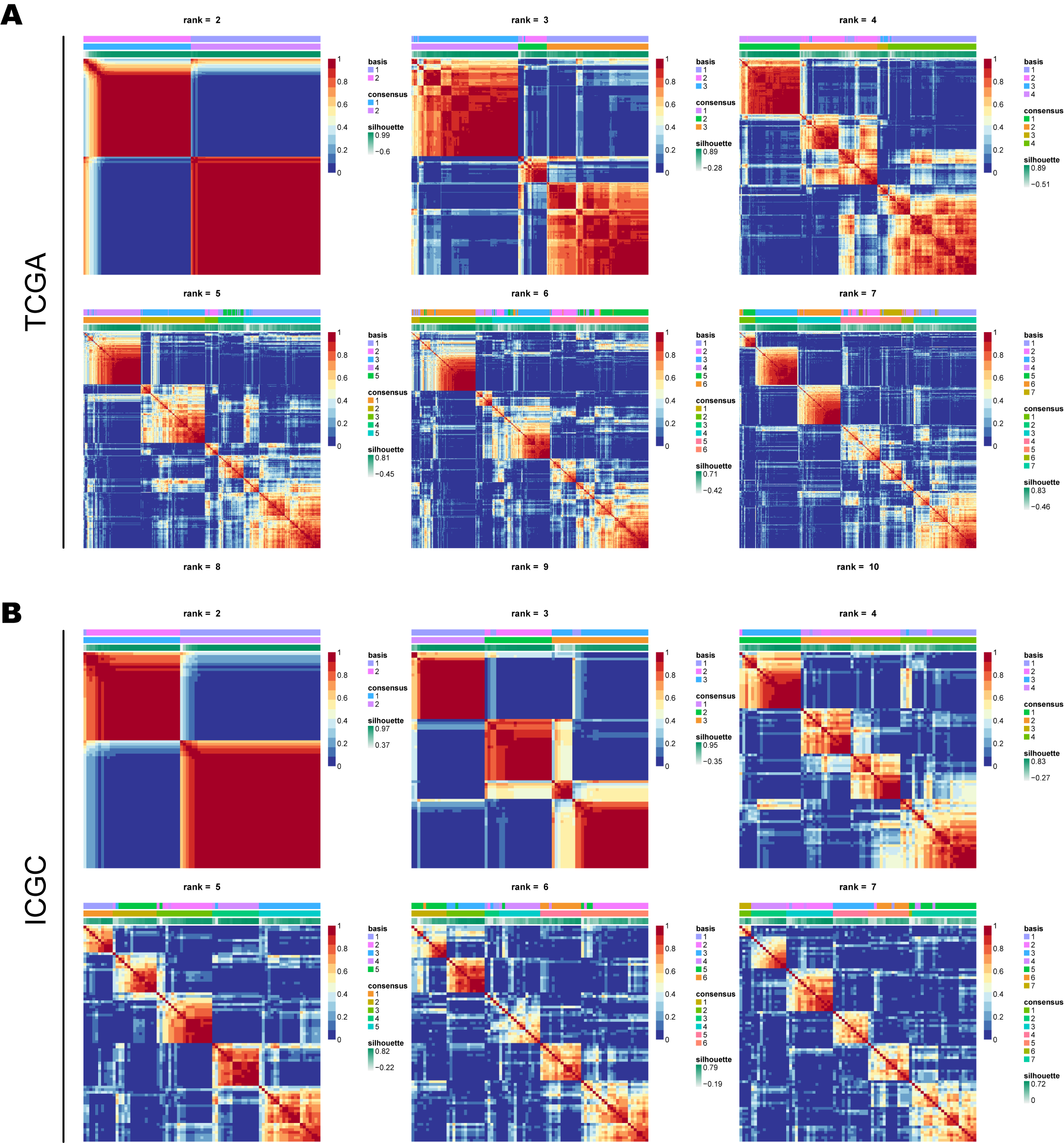


**Figure S1.** The consensus matrix heatmap of TCGA and ICGC cohort for k = 2-7 was shown.


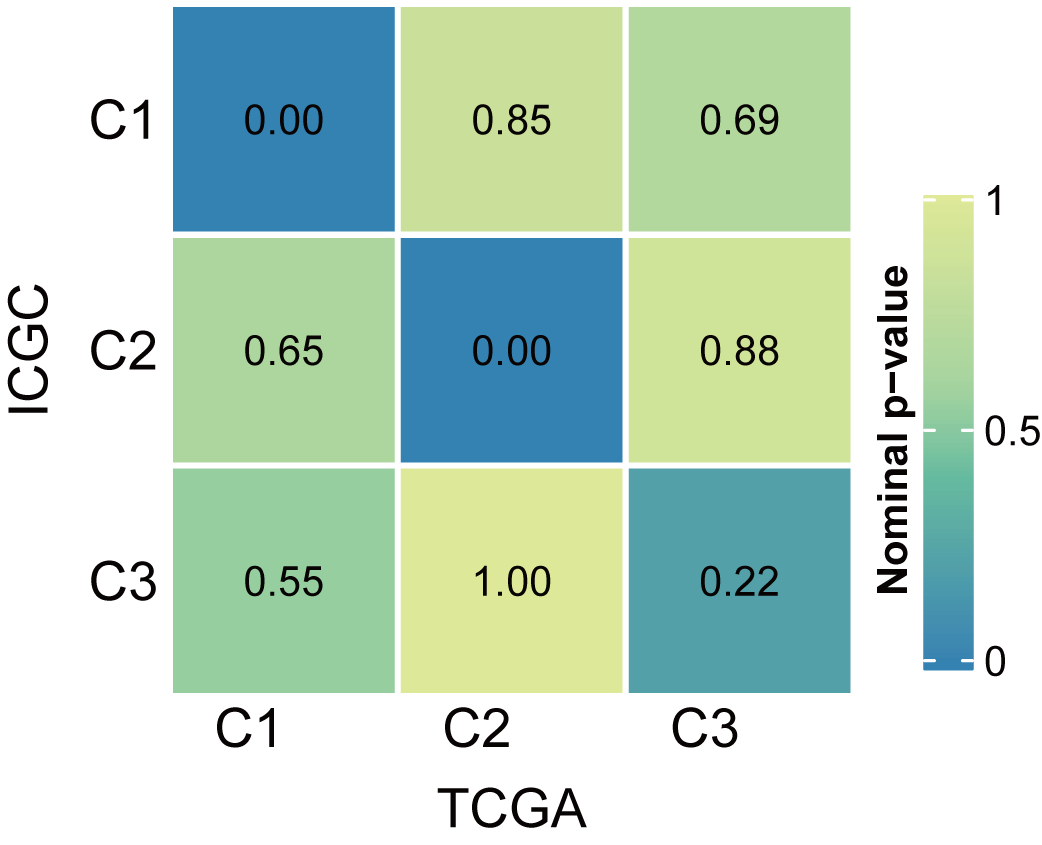


**Figure S2.** Correlation between subtypes identified in the TCGA and ICGC cohorts using SubMap analysis. More blue represented the more significantly correlation. The number represented the *P*-value of correlation.


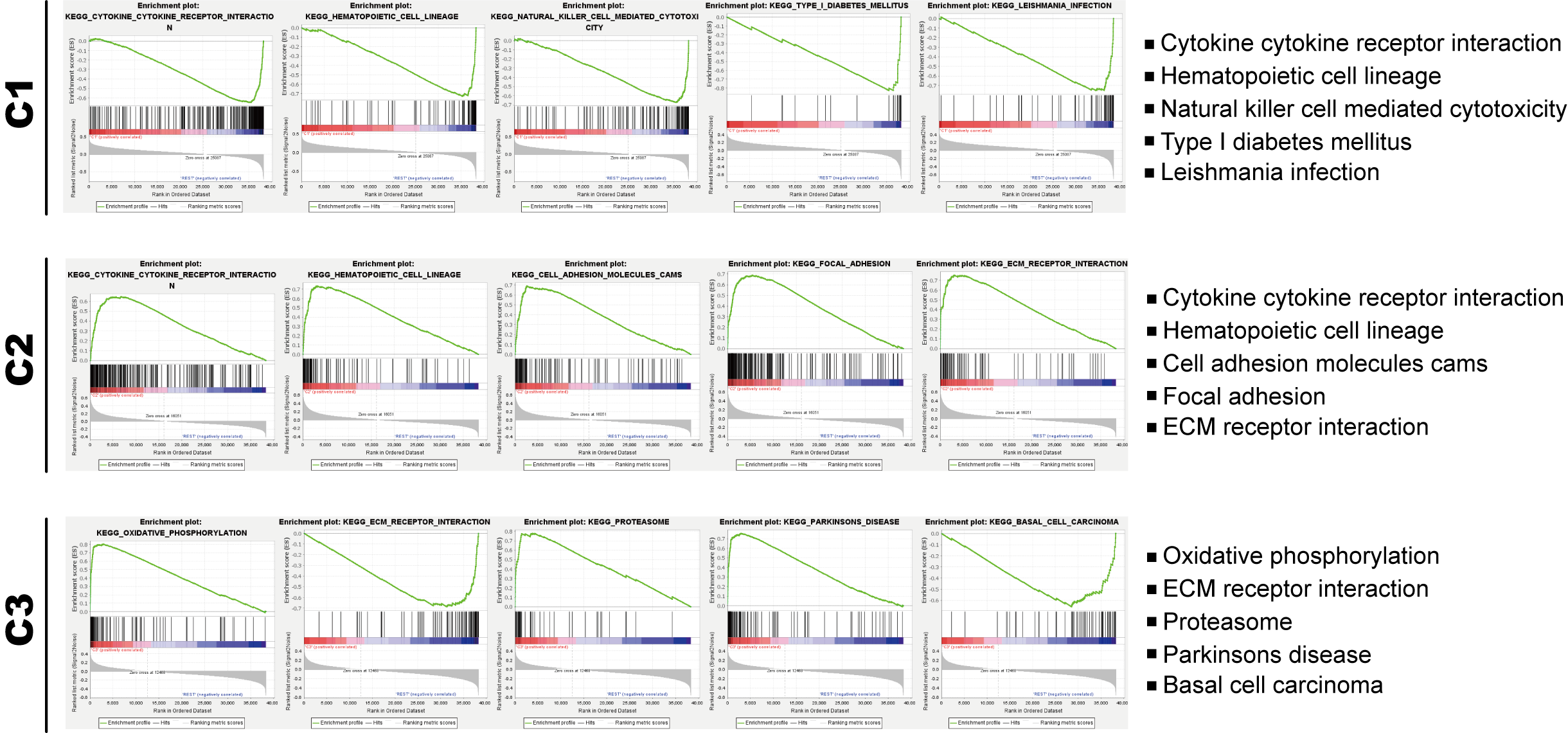


**Figure S3.** The top 5 enriched KEGG pathways between each subtype and the rest subtypes based on raw counts matrix using GSEA analysis.


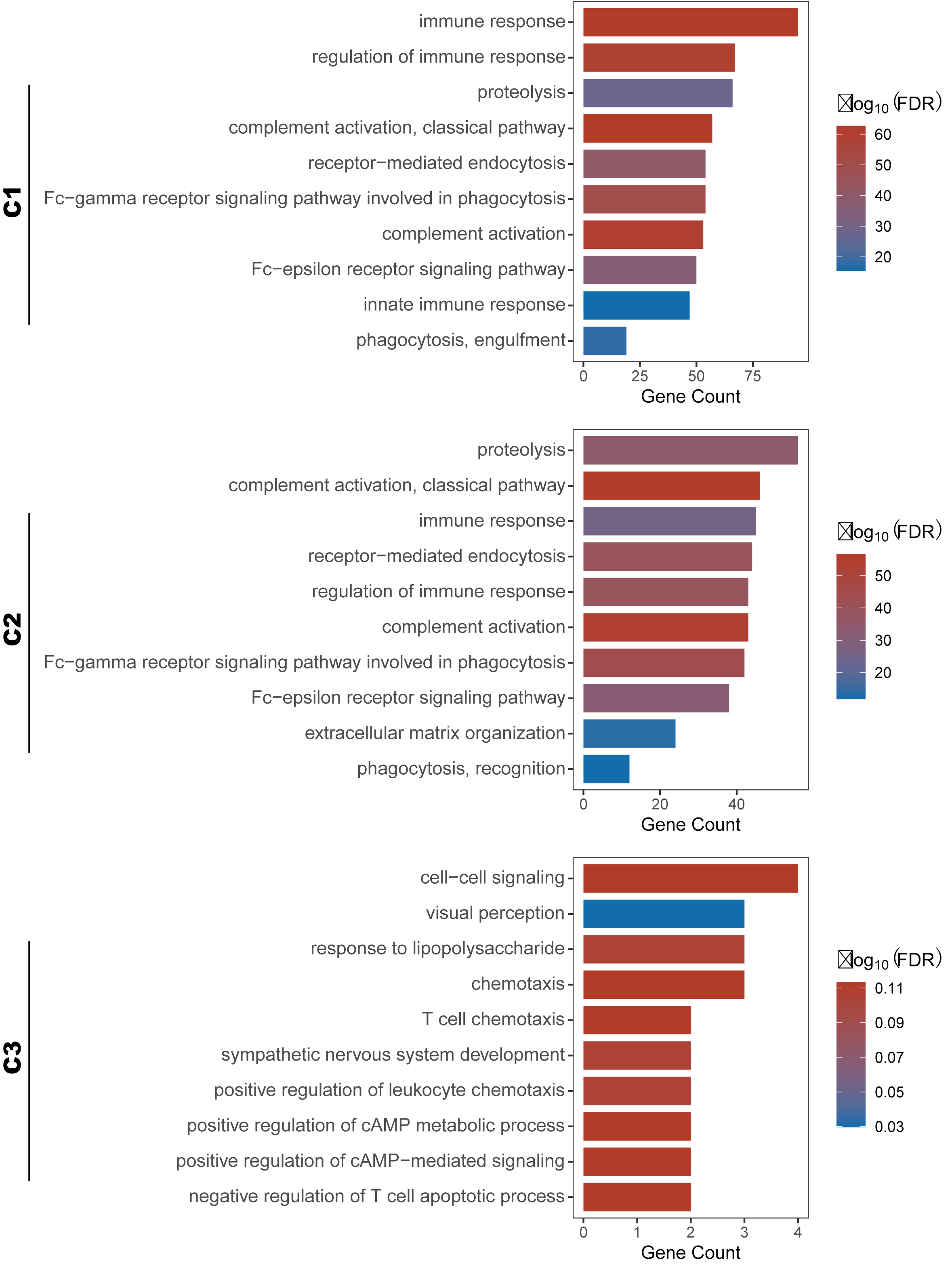


**Figure S4.** The top 10 enriched GO BP between each subtype and the rest subtypes based on differential genes using David analysis.


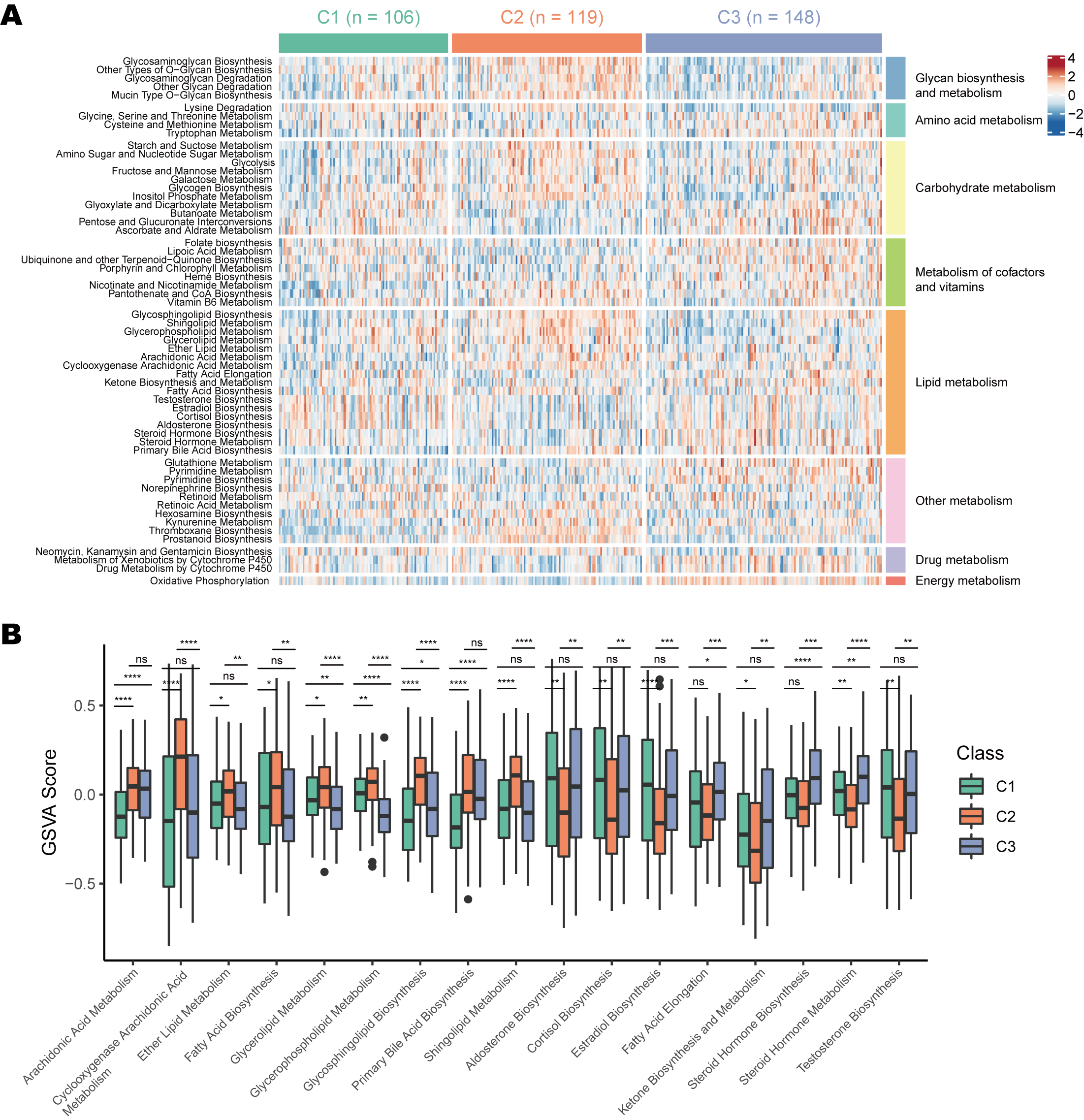


**Figure S5.** The association between metabolism pathway signatures and the TCGA-OV subtypes. (A) Heatmap of the specific metabolism-associated pathway signatures in C2 subtype. (B) Boxplot of the signatures score for OC lipid metabolism relevant signatures between 3 subtypes. The statistical difference was compared by wilcox.test, and adjusted by “holm” method (ns represents no significance, **P* <0.05, ***P* <0.01, ****P* <0.001, *****P* <0.0001).


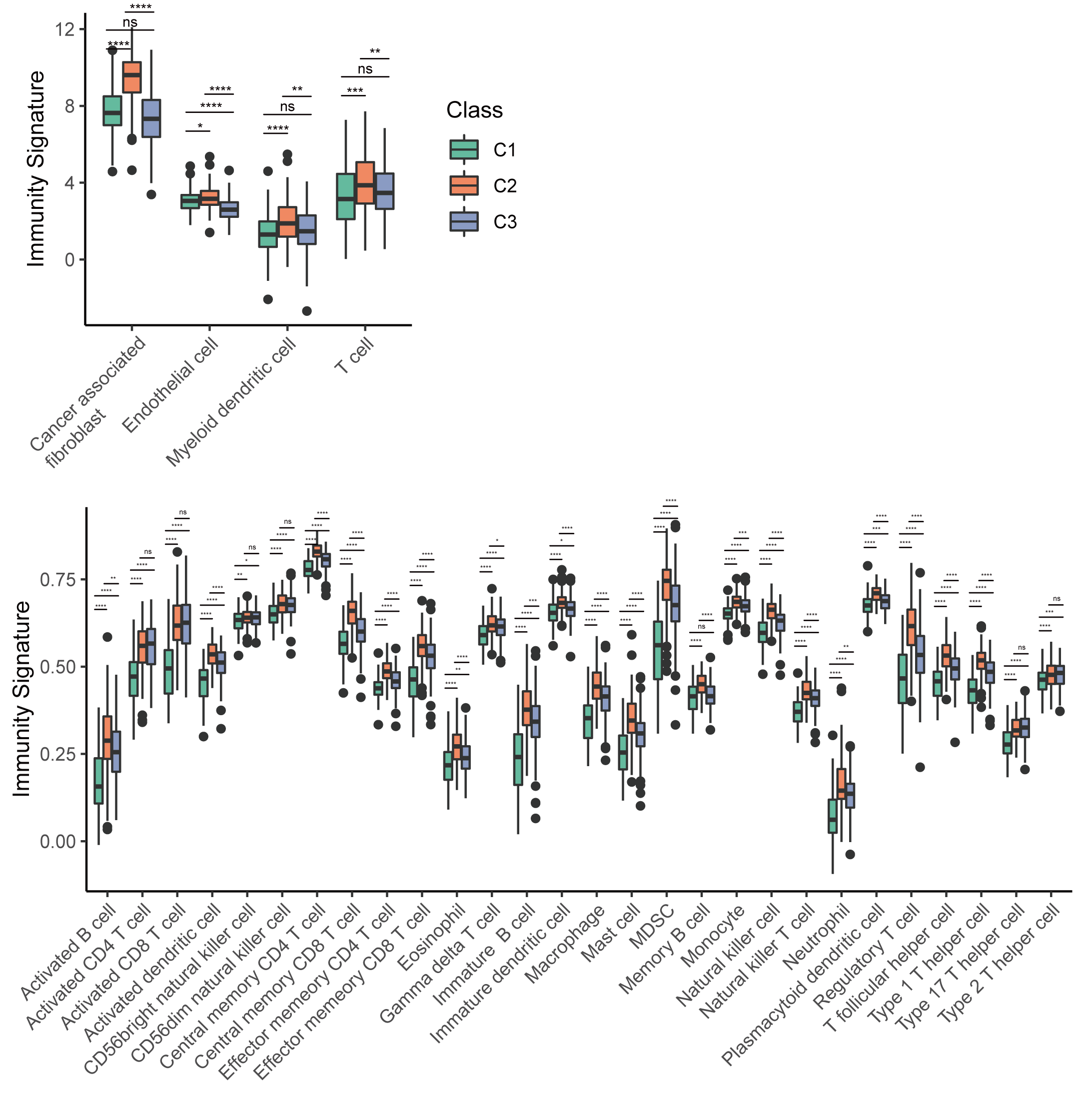


**Figure S6.** Boxplot of the abundance of immune and stromal cell populations between 3 subtypes. The statistical difference was compared by wilcox.test, and adjusted by “holm” method (ns represents no significance, **P* <0.05, ***P* <0.01, ****P* <0.001, *****P* <0.0001).


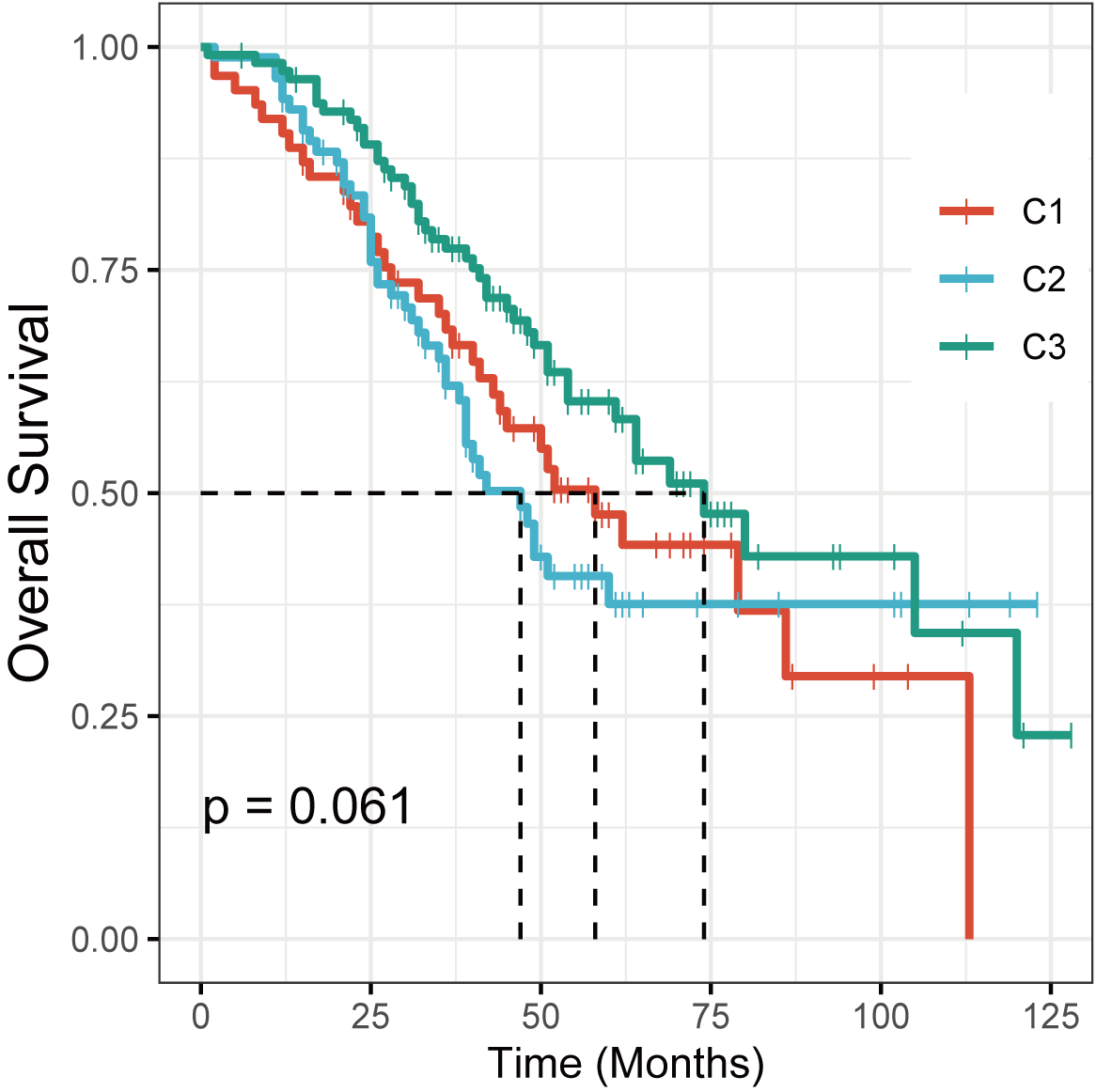


**Figure S7.** KM curves showed prognostic relationship of predicted 3 subtypes in GSE32062 cohort; the P-value was calculated using the log-rank test, by comparing the overall survival of 3 subtypes.
